# Aging as a Context for the Role of Inflammation in Depressive Symptoms

**DOI:** 10.3389/fpsyt.2020.605347

**Published:** 2021-01-18

**Authors:** Kelci Straka, Mai-Lan Tran, Summer Millwood, James Swanson, Kate Ryan Kuhlman

**Affiliations:** ^1^Department of Psychological Science, University of California, Irvine, Irvine, CA, United States; ^2^Cousins Center for Psychoneuroimmunology, Semel Institute for Neuroscience and Human Behavior, University of California, Los Angeles, Los Angeles, CA, Unites States; ^3^Interdisciplinary Institute for Salivary Bioscience Research, University of California, Irvine, Irvine, CA, United States

**Keywords:** aging, depression, inflammation, lifespan, somatic complaints, anhedonia, immune system

## Abstract

Inflammation has been implicated in the pathogenesis and maintenance of depressive symptoms. The role of inflammation in depressive symptomatology may be complex, varying within endophenotypes and across the lifespan. Aging is associated with myriad changes in the structure and function of the brain. Yet, little attention has been given to the role of inflammation in depressive symptoms within a lifespan developmental framework. In this study, we examined whether the association between inflammation and depressive symptom domains varied by age. Participants were a community sample of individuals (*N* = 2,077, Range = 30–84) who participated in the Biomarker projects of the MIDUS2, MIDUS Refresher, or the MIDJA study. Inflammation was indexed by two inflammatory markers consistently implicated in depressed individuals, interleukin 6 (IL-6) and C-reactive protein (CRP), measured in blood. Depressive symptom domains, including depressed affect, anhedonia, somatic complaints, and interpersonal problems, were reported *via* the Center for Epidemiologic Studies—Depression Scale (CES-D). Inflammatory markers were associated with more somatic complaints, more interpersonal problems, and less anhedonia. Age moderated the relationship between inflammatory markers and two depressive symptom subscales. Specifically, the positive association between inflammation and somatic complaints and the negative association between inflammation and anhedonia increased with age. These observations offer preliminary evidence from a large community sample that aging may be an important context for the role of inflammatory signaling in different aspects of psychological and behavioral well-being.

## Introduction

Depression affects 264 million people globally every year including millions of Americans (1). Major depressive disorder (MDD) causes significant loss of productivity in the workplace and is the sixth costliest health condition in the U.S. (2–4). Indeed, the economic burden of depression is estimated at $210.5 billion in the U.S. alone (5). Depressed persons are also 20.9 times more likely to die by suicide and two times more likely to die prematurely due to other causes (6). Yet, depression treatments with the most empirical support appear to have modest effectiveness (7). Thus, a better understanding of the factors that lead to depressive symptoms is needed in order to develop more effective treatments for this burdensome disease.

Inflammation has been linked to depressive symptoms in studies examining both exogenously-induced and naturally occurring inflammation (8, 9). Inflammation refers to the production of soluble proteins called cytokines by immune cells in response to potential threats (10). Inflammatory proteins play a key role in the communication between the immune system and other systems such as the central nervous system, which is the putative pathway that may lead to depressive symptoms (11–13). While short-term inflammatory activity serves a functional role in preventing disease, chronic systemic inflammation has been linked with increased morbidity and mortality (14–16). Indeed, the two most common markers of systemic inflammation, C-Reactive Protein (CRP) and interleukin (IL) 6, are consistently elevated among individuals with major depressive disorder relative to controls (12). Additionally, several studies have found that elevated inflammatory markers precede depressive episodes (17–19).

Depression is a heterogeneous disorder comprised of several endophenotypes, or domains of possible impairment that may be biologically distinct from one another (20, 21). Endophenotypes provide an intermediary link between genes and the visible consequence of those genes (phenotypes) (20). Common endophenotypes observed in individuals with depression are: negative emotionality and reactivity, impaired reward function, impaired learning and memory, impaired executive function, psychomotor slowing, and exaggerated stress sensitivity (21, 22). These depressive endophenotypes correspond to several depressive symptom domains: fatigue and psychomotor slowing correspond to somatic complaints, negative emotionality and reactivity correspond to depressed mood, and impaired reward function corresponds to anhedonia (22). Furthermore, previous research has shown that exogenous activation of the immune system leads to exaggerated reactivity to negative information, altered reward activity, and somatic complaints (22).

Signaling from the immune system to the brain *via* inflammation may be the biological basis of several depressive symptom domains (8, 9). Indeed, inflammation has been linked with somatic complaints (23–32), depressed affect (30, 33, 34), anhedonia (27, 35, 36), and interpersonal problems including feelings of social disconnection and troubled relationships (37–40). However, the observed association between inflammation and each of these symptom domains has been mixed, suggesting differential associations in older adults relative to healthy, young adults. Notably, a longitudinal study of older adults observed that sustained elevations in inflammation, as measured by CRP, were more robustly linked to somatic complaints than cognitive-affective depressive symptoms over time (41). Furthermore, the association between inflammation and positive affect, depressed affect or interpersonal problems has been less robust or not observed among samples in middle and later adulthood [c.f. (42–45)] In summary, inflammation has been linked to several commonly observed phenotypes in depression, though the literature reflects many apparent discrepancies involving the role of age.

There is a complex phenomenon that occurs during the aging process. Systemic inflammation increases with age (46). Yet, depressive symptoms follow a nonlinear trajectory; they are highest among young adults, decline across middle adulthood, then rise again among the oldest old (47). Indeed, older adults (ages 60+ years) experience relatively high rates of subthreshold depressive symptoms that are associated with clinically meaningful functional impairment (48–50). Suicide rates are also consistently higher in both late middle age (ages 45–59) and in older age (age 80 and above) than among young adults [see also (51) for a special issue on this topic; (52, 53)]. Despite this, rates of diagnosed depression tend to decline across middle adulthood (14, 54). Indeed, American adults aged 50 and over are 1.6 times less likely to be diagnosed with depression than adults aged 26–49 and 2.8 times less likely to be diagnosed with depression than young adults under age 25 (55). Thus, depression may be underdiagnosed in older populations, possibly due to differential symptom presentation with age. A plausible explanation for this phenomenon is that age modifies the sensitivity of the central nervous system to inflammatory signaling, such that immune activation leads to different depressive endophenotypes as the brain changes.

While systemic inflammation increases with age, the sensitivity of different neural circuits and their functional outcomes due to inflammation may vary across the lifespan. Indeed, systemic inflammation increases with age, leading to a heightened inflammatory state even in healthy older adults (46). There are also normative decreases in some brain structures and functions that begin as early as age 20 (56–58). Our central hypothesis is that changes to the structure and function of the brain that occur with age [see (56) for review] may alter the influence of inflammatory signaling on psychological phenomena, including many depressive symptoms. In the current study, we aimed to explore the potential moderating effect of age on the relationship between inflammation and depression endophenotypes. We expected that there would be a positive association between inflammation and somatic complaints, depressed affect, anhedonia, and interpersonal problems. Given the observations in the literature that older adults are more susceptible to somatic complaints while also less reactive to negative stimuli, we expected that the links between inflammation and somatic complaints would increase with age, while the links between inflammation and affective symptoms (depressed affect and anhedonia) would decrease with age (41). We did not formulate an a priori hypothesis about the moderating effect of age on the link between inflammation and interpersonal symptoms due to a lack of consensus in the existing literature.

## Methods

### Participants

The present study includes data from a community sample of 2,077 individuals (54.3% female, 66.8% currently married; 90.3% Caucasian) between the ages of 30 and 84 who participated in the Biomarker projects from the 2nd wave of the Midlife in the United States (MIDUS2) (*n* = 1,255), the refresher cohort of the Midlife in the United States (MIDUS-R) (*n* = 863), and the Midlife in Japan (MIDJA) (*n* = 382) studies. The MIDUS-R and MIDJA studies were conducted to increase the diversity of the overall MIDUS sample in order to allow tests of hypotheses about the role of psychosocial factors in the health (broadly defined) of mid- and later-life adults across multiple cultural contexts. The MIDUS2 and MIDUS-R samples were predominantly white, 93 and 82%, respectively, and more than 1/3 of the sample had attained a bachelor's degree or higher, while the MIDJA sample was uniformly Japanese and just under 1/3 of the sample attained a bachelor's degree or higher. Of the 2,500 participants who participated in one of these three studies, 2,428 (97.1%) had complete data for all of our primary variables and covariates. Consistent with consensus guidelines in research involving inflammatory markers (59, 60), as well as previous studies using data from MIDUS [e.g., (61)], 251 individuals were excluded for current smoking, 84 were excluded for likely infection (CRP > 10 μg/mL), and 16 were excluded for both smoking and likely infection. Current smokers were excluded due to tobacco smoking being linked to increases in inflammatory markers such as IL-6 and CRP (59).

MIDUS2 participants originated from the Midlife in the United States study conducted from 1995 to 1996 and were recruited *via* random-digit dialing of potential respondents ages 25–75 across the 48 contiguous states. A diverse population of 7,108 participants included the respondents, eligible siblings, and twins. The MIDUS2 Biomarker project [2004–2009; (62)] included a total of 1,255 participants that stayed overnight at one of three general clinical research centers (GCRC) at the University of California Los Angeles (UCLA), the University of Wisconsin (UW), or Georgetown University (GU).

MIDUS-R participants were from a refresher study [MIDUS Refresher; (63)] conducted in 2011– 2014 to replenish the original MIDUS cohort with a new sample containing 3,577 participants ages 24–75. The MIDUS-R Biomarker project [2012–2016; (64)] included a total of 863 participants that stayed overnight at one of three GCRCs.

MIDJA participants were from the Midlife in Japan study [MIDJA; (65)], a sister study to MIDUS in 2008 comparing the psychosocial factors in the health of adults between Japan and the U.S. Participants ages 30–79 years old were randomly sampled from the 23 wards of Tokyo and were recruited *via* a “deliver-and-pick-up” method. Our analyses used a subset of participants from the MIDJA study that agreed to partake in the Biomarker project in 2009–2010. The MIDJA Biomarker project (66) included 382 participants that stayed overnight at a medical clinic near the University of Tokyo for biological assessments. MIDJA participants completed the same questionnaire battery as the MIDUS sample which was translated into Japanese.

### Procedures

All study procedures for all included projects were approved by the Institutional Review Board at each site prior to data collection. Participation in the MIDUS2 and MIDUS-R Biomarker projects required a 2-day stay at one of the three GCRC's/CRU's. MIDUS2 participants completed a self-report questionnaire for demographics and psychosocial assessments. The second day involved a fasting blood collection at 6:30–7:00 a.m., as well as a physical exam. MIDUS-R participants underwent the same protocol, except participants completed the physical exam on the first day. Blood samples were processed at the GCRC/CRU, and then frozen and shipped to the MIDUS Biocore Labs at the University of Wisconsin and the University of Vermont for assay. Serum IL-6 was assayed at the University of Wisconsin, and plasma CRP was assayed at the University of Vermont.

Participants in the MIDJA Biomarker project completed their biological assessments during the day at a medical clinic near the University of Tokyo. At the clinic, a physical exam was administered, and then a non-fasting blood draw was completed. Blood samples were immediately processed at the University of Tokyo, sent to Syowa Medical services to be frozen until they were shipped to the Biocore lab in Madison, WI, USA to be assayed under the same protocols as the MIDUS2 and MIDUS-R Biomarker projects. Upon leaving the clinic, participants were given an at-home assessment packet that included psychosocial measures such as the CES-D. Once completed, the packet was mailed to Tokyo Women's Christian University.

### Measures

#### Inflammation

Inflammation was measured *via* concentrations of CRP in plasma and IL-6 in serum. Plasma CRP was measured *via* a BNII nephelometer (Dade Behring Inc., Deerfield, IL) with a particle enhanced immunonepholometric assay. The range of detection for this assay was 0.175–1,100 μg/mL. CRP samples that fell outside the assay range were re-assayed *via* immunoelectrochemiluminescence using a high-sensitivity assay kit (Meso Scale Diagnostics #K151STG). Intra-assay CV ranged from 2.2 to 4.4% and inter-assay CV ranged from 2.1 to 5.7%. Serum IL-6 was assessed using a Quantikine high-sensitivity enzyme-linked immunosorbent assay kit (R & D Systems, Minneapolis, MN). The range of detection for this assay was 0.156–10 pg/mL. Intra-assay coefficient of variability (CV) was 3.2% and inter-assay CV was 12.3%.

#### Depressive Symptoms

Depressive symptoms were measured using the well-validated and widely used Center for Epidemiologic Studies—Depression Scale (CES-D) (67, 68). This 20-item questionnaire contains four subscales: depressed affect (7 items), positive affect (recoded as anhedonia; 4 items), somatic complaints (7 items), and interpersonal problems (2 items). For each item, participants were asked to rate how often they felt a certain way during the past week (e.g., “I felt depressed” and “I was bothered by things that usually don't bother me.”). Respondents rated on a 4-point scale ranging from 0 (rarely or none of the time) to 3 (most or all of the time), and responses were summed to create total and subscale scores. The positive affect subscale was then reverse coded to reflect anhedonia. Total scores could range from 0 to 60, with higher scores indicating more depressive symptoms, and scores >15 indicating a likely major depressive episode. CES-D scores have high construct validity in older adults (69, 70), and the Japanese version of the CES-D has shown specificity and external validity (71, 72), with a similar factor structure (73). Internal reliabilities of all subscales were good in this sample, α = 0.82–0.89.

### Data Analysis

All continuous variables were examined for normality and heteroscedacity. IL-6 and CRP were both transformed using the natural log transformation. Based upon best practices in research using inflammatory biomarkers (59), all analyses covaried for sex and body mass index (BMI). All analyses also covaried for the study from which the data was drawn (MIDUS2, MIDUS-R, or MIDJA) because the method of biomarker collection differed between the MIDUS and MIDJA samples, and there were significant differences between participants in MIDUS2, MIDUS-R, and MIDJA samples in age, *F*_(2, 2,074)_ = 23.81, *p* < 0.001, BMI, *F*_(2, 2,074)_ = 202.07, *p* < 0.001, IL-6, *F*_(2, 2,074)_ = 112.33, *p* < 0.001, CRP, *F*_(2, 2,074)_ = 243.38, *p* < 0.001, and depressive symptoms, *F*_(2, 2,074)_ = 59.56, *p* < 0.001.

To determine the main effect of CRP, IL-6, and age on depressive symptoms, we conducted multiple regression models predicting depressed affect, anhedonia, somatic complaints, and interpersonal problems separately from CRP and IL-6, as well as age, while accounting for our key covariates.

To determine whether age moderated the association between CRP or IL-6 and depressive symptoms, we used the PROCESS Macro for SPSS to estimate the interaction between CRP or IL-6 and age as a predictor of each depressive symptom domain (74), as well as to compute the association between CRP, IL-6, and depressive symptoms for individuals ±1SD from the mean age in our sample. All predictors were mean-centered for analyses, and all interaction models included key covariates. In models where the reliability of the estimated interaction was <0.10, we used the Johnson-Neyman method to determine the age at which the pattern of association between CRP or IL-6 and depressive symptoms reached significance. All *p*-values are reported for transparency and to facilitate comparison with other studies, but a *p*-value <0.05 is considered statistically reliable, and a *p*-value <0.007 corrected for multiple comparisons (75, 76).

## Results

Depressive symptoms in the sample varied widely, such that total depressive symptom scores ranged from 0 to 55, and 15.6% (*n* = 324) of participants exceeded the clinical threshold on the CES-D, indicating possible Major Depressive Disorder. Table 1 provides descriptive information about inflammatory markers and depressive symptoms by age quartiles. Table 2 provides descriptive statistics for all key study variables and the bivariate correlations between them. As expected, both inflammatory markers were correlated with several depressive outcomes. CRP was associated with more somatic complaints and less anhedonia. Higher IL-6 was associated with more somatic complaints, less anhedonia, and more interpersonal problems.

**Table 1:** Inflammatory markers and depressive symptom domains by age (Quartiles).

**Quartile**	**1**	**2**	**3**	**4**
**Age (years)**	**25–43**	**44–53**	**54–63**	**64–84**
*n*	515	522	509	531
**Inflammation**				
CRP (M ± SD)	1.83 (2.20)	1.88 (2.07)	1.92 (2.13)	1.84 (1.98)
IL-6 (M ± SD)	1.89 (1.82)	2.07 (2.01)	2.61 (2.45)	3.23 (2.91)
**Depressive symptoms**				
% Clinically elevated	21.0	19.0	13.9	8.7
Depressed affect (M ± SD)	2.30 (3.14)	2.24 (3.20)	1.49 (2.55)	1.16 (2.19)
Anhedonia (M ± SD)	3.71 (3.15)	3.22 (2.98)	3.28 (3.27)	3.39 (3.42)
Somatic complaints (M ± SD)	3.82 (3.35)	3.54 (3.11)	2.98 (3.27)	2.83 (2.61)
Interpersonal problems (M ± SD)	0.58 (0.95)	0.47 (0.87)	0.31 (0.76)	0.25 (0.63)

**Table 2:** Descriptive statistics for key study variables and bivariate associations between them.

	***M*(SD)**	**Range**	**Correlations**						
			**1**.	**2**.	**3**.	**4**.	**5**.	**6**.	**7**.
1. Age	53.80 (12.98)	25–84	1.00						
2. BMI	28.43 (6.61)	14.99–77.58	0.01	1.00					
**Inflammatory markers**									
3. CRP (μg/mL)	1.86 (2.09)	0.02–10.00	0.06^**^	0.53^***^	1.00				
4. IL-6 (pg/mL)	2.45 (2.40)	0.03–23.00	0.31^***^	0.44^***^	0.56^***^	1.00			
**Depressive symptoms (CESD)**									
5. Depressed affect	1.80 (2.84)	0–20	−0.17^***^	0.08^***^	0.06^**^	0.01	1.00		
6. Anhedonia	3.40 (3.22)	0–12	−0.02	−0.14^***^	−0.19^***^	−0.12^***^	−0.37^***^	1.00	
7. Somatic complaints	3.29 (3.04)	0–18	−0.13^***^	0.12^***^	0.11^***^	0.07^**^	0.64^***^	0.22^***^	1.00
8. Interpersonal problems	0.40 (0.82)	0–6	−0.17^***^	0.07^**^	0.04	0.02	0.42^***^	0.19^***^	0.41^***^

** *p < 0.01*,

*** *p < 0.001*;

*BMI, body mass index; IL, interleukin; CRP, C-reactive protein; CESD, Center for Epidemiological Studies—Depression scale*.

### Inflammatory Markers as a Predictor of Depressive Symptoms

After adjusting for sex, BMI, and study, CRP was associated with more somatic complaints, *b* = 0.18, *SE* = 0.07, *p* = 0.008, *R*^2^ = 0.04, *F*_(5, 2,071)_ = 16.66, *p* < 0.001, and less anhedonia, *b* = −0.43, *SE* = 0.07, *p* < 0.001, *R*^2^ = 0.04, *F*_(5, 2,071)_ = 18.90, *p* < 0.001. CRP was not associated with depressed affect, *b* = 0.07, *SE* = 0.06, *p* = 0.25, *R*^2^ = 0.04, *F*_(5, 2,071)_ = 17.69, *p* < 0.001, or interpersonal problems, *b* = 0.02, *SE* = 0.02, *p* = 0.27, *R*^2^ = 0.03, *F*_(5, 2,071)_ = 13.88, *p* < 0.001. After adjusting for sex, BMI, and study, IL-6 was associated with more somatic complaints, *b* = 0.32, *SE* = 0.10, *p* = 0.002, *R*^2^ = 0.04, *F*_(5, 2,071)_ = 17.26, *p* < 0.001, less anhedonia, *b* = −0.28, *SE* = 0.11, *p* = 0.01, *R*^2^ = 0.03, *F*_(5, 2,071)_ = 12.54, *p* < 0.001, and greater interpersonal problems, *b* = 0.06, *SE* = 0.03, *p* = 0.018, *R*^2^ = 0.03, *F*_(5, 2,071)_ = 14.79, *p* < 0.001. IL-6 was not associated with depressed affect, *b* = 0.14, *SE* = 0.09, *p* = 0.13, *R*^2^ = 0.04, *F*_(5, 2,071)_ = 17.90, *p* < 0.001.

### Age as a Moderator of Inflammation on Depressive Symptoms

**Figure 1:**
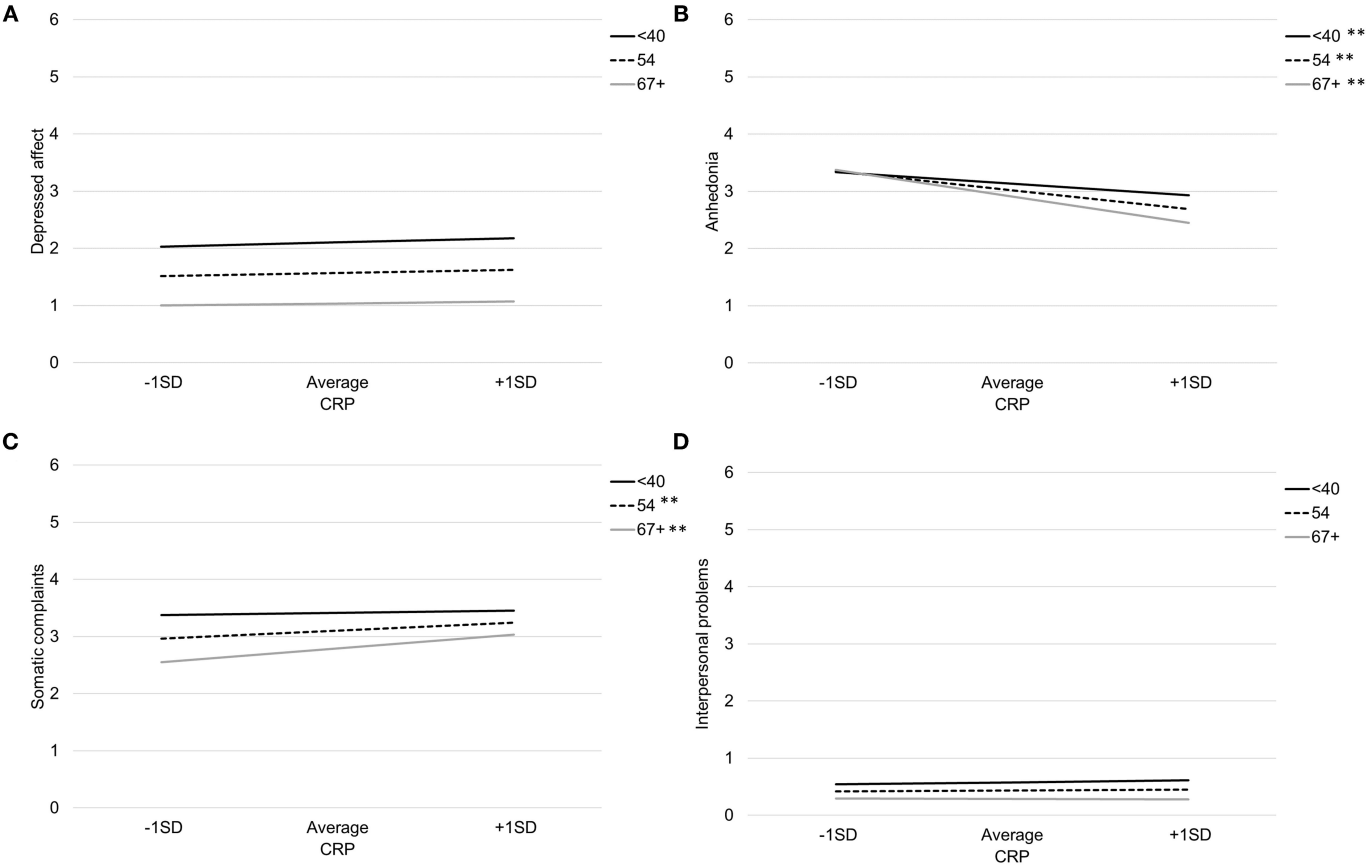
Association between CRP and depressed affect **(A)**, anhedonia **(B)**, somatic complaints **(C)**, and interpersonal problems **(D)** by age. Note: **p < 0.007, *p < 0.05.

Table 3 provides coefficient estimates from the interaction models predicting depressive symptom domains as a function of CRP, age, their interaction, and our covariates (sex, BMI, and study). Figure 1 illustrates the association between CRP and each depressive symptom domain as a function of age.

**Table 3:** Estimated coefficients predicting depressive symptoms and phenotypes from CRP, age, and their interaction.

	**Depressed affect**		**Anhedonia**		**Somatic complaints**		**Interpersonal problems**	
***R*^**2**^**	0.04^***^		0.05^***^		0.04^***^		0.03^***^	
**Predictor**	***b (SE)***	***p***	***b (SE)***	***p***	***b (SE)***	***p***	***b (SE)***	***p***
Intercept	1.53 (0.11)	<0.001	3.27 (0.13)	<0.001	3.00 (0.12)	<0.001	0.42 (0.03)	<0.001
CRP	0.07 (0.06)	0.25	−0.43 (0.07)	<0.001	0.18 (0.07)	0.008	0.02 (0.02)	0.28
Age	−0.04 (0.005)	<0.001	−0.001 (0.005)	0.83	−0.03 (0.005)	<0.001	−0.01 (0.001)	<0.001
CRP x age	−0.002 (0.004)	0.55	−0.013 (0.005)	0.006	0.01 (0.004)	0.01	−0.002 (0.001)	0.20
**Covariates**								
Female	0.44 (0.12)	<0.001	−0.05 (0.14)	0.72	0.37 (0.13)	0.006	−0.03 (0.04)	0.38
Sub-study	0.05 (0.07)	0.50	0.21 (0.08)	0.007	0.09 (0.07)	0.22	0.01 (0.02)	0.58
BMI	0.03 (0.01)	0.01	−0.03 (0.01)	0.006	0.04 (0.01)	<0.001	0.005 (0.003)	0.11

*** *p < 0.001*.

Age significantly moderated the association between CRP and both somatic complaints and anhedonia. There was a significant interaction between CRP and age when predicting somatic complaints, *b* = 0.011, *SE* = 0.004, *p* = 0.01. Specifically, CRP was associated with more somatic complaints among older participants (>66 years), *b* = 0.32, *SE* = 0.09, *p* < 0.001, and average aged participants (41–66 years), *b* = 0.18, *SE* = 0.07, *p* = 0.006, but not younger participants (<41 years), *b* = 0.04, *SE* = 0.09, *p* = 0.67. Using the Johnson-Neyman technique, the association between CRP and greater somatic complaints was significant and positive after about age 51. There was also a significant interaction between CRP and age when predicting anhedonia, *b* = −0.013, *SE* = 0.005, *p* = *0*.006. Specifically, the negative association between CRP and anhedonia was stronger with increasing participant age; younger participants (<41 years) *b* = −0.27, *SE* = 0.09, *p* = 0.003, average aged participants (41–66 years) *b* = −0.43, *SE* = 0.07, *p* < 0.001, and older participants (>66 years) *b* = −0.60, *SE* = 0.09, *p* < 0.001. Using the Johnson-Neyman technique, the association between CRP and anhedonia emerged after about age 37 and increased in magnitude throughout the lifespan.

**Figure 2:**
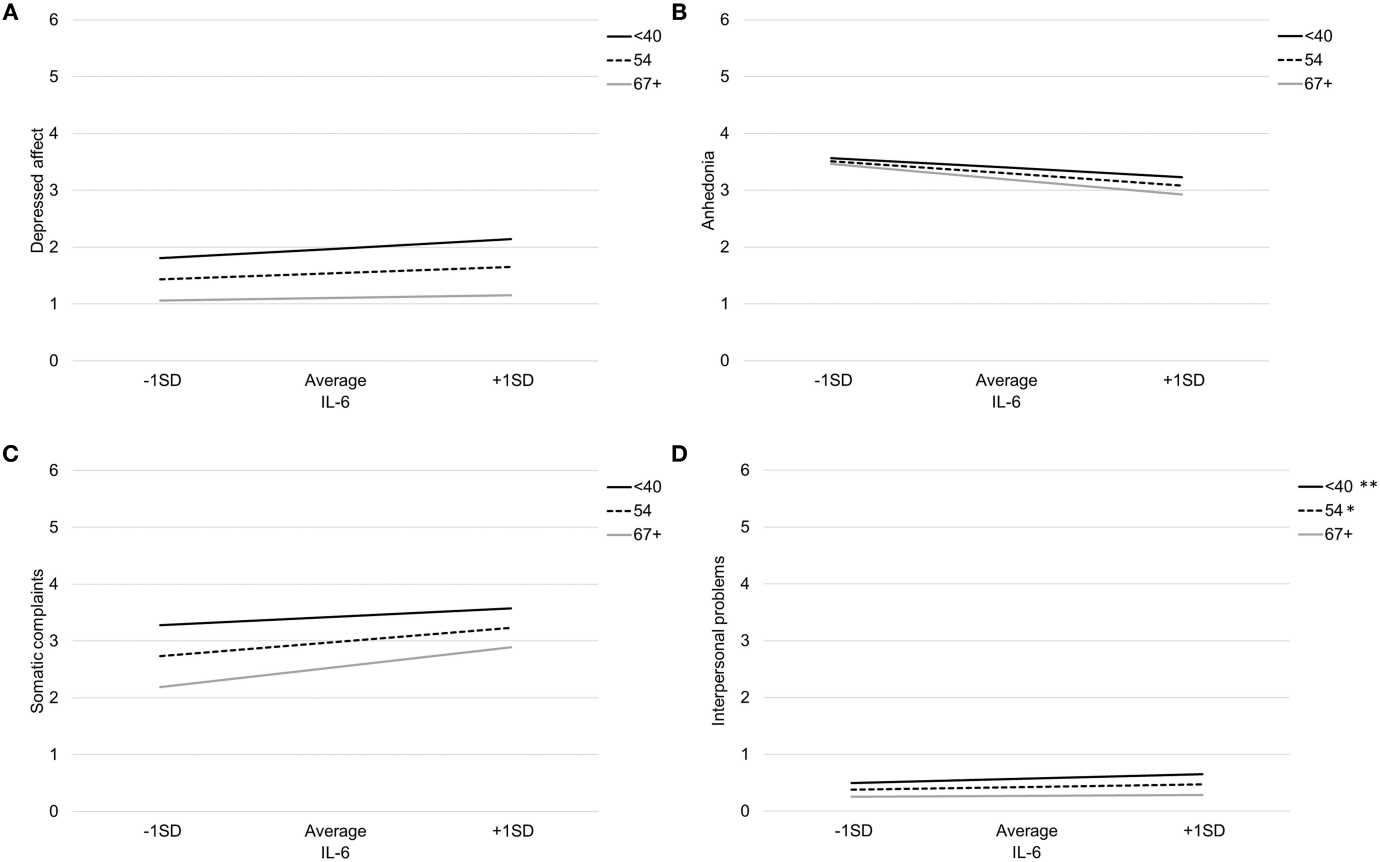
Association between IL-6 and depressed affect **(A)**, anhedonia **(B)**, somatic complaints **(C)**, and interpersonal problems **(D)** by age. Note: **p < 0.007, *p < 0.05.

Table 4 provides coefficient estimates from the interaction models predicting depressive symptom domains as a function of IL-6, age, their interaction, and our covariates (sex, BMI, and study). Figure 2 illustrates the association between IL-6 and each depressive symptom domain as a function of age. Age did not significantly moderate the association between IL-6 and any symptom domains.

**Table 4:** Estimated coefficients predicting depressive symptoms and phenotypes from IL-6, age, and their interaction.

	**Depressed affect**		**Anhedonia**		**Somatic complaints**		**Interpersonal problems**	
***R*^**2**^**	0.04^***^		0.03^***^		0.04^***^		0.036^***^	
**Predictor**	***b (SE)***	***p***	***b (SE)***	***p***	***b (SE)***	***p***	***b (SE)***	***p***
Intercept	1.46 (0.12)	<0.001	3.46 (0.14)	<0.001	2.80 (0.13)	<0.001	0.39(0.04)	<0.001
IL-6	0.14 (0.09)	0.13	−0.28 (0.11)	0.01	0.32 (0.10)	0.0015	0.06 (0.03)	0.019
Age	−0.03 (0.01)	<0.001	0.005 (0.007)	0.50	−0.04 (0.01)	<0.001	−0.01 (0.002)	<0.001
IL-6 x age	−0.006 (0.006)	0.32	−0.005 (0.01)	0.48	0.01 (0.01)	0.10	−0.003 (0.002)	0.06
**Covariates**								
Female	0.44 (0.12)	<0.001	−0.14 (0.14)	0.31	0.40 (0.13)	0.002	−0.03 (0.04)	0.37
Sub-study	0.05 (0.07)	0.46	0.24 (0.08)	0.002	0.07 (0.07)	0.34	0.01 (0.02)	0.47
BMI	0.03 (0.01)	0.01	−0.06 (0.01)	<0.001	0.04 (0.01)	<0.001	0.004 (0.003)	0.26

*** *p < 0.001*.

There was a non-significant interaction between IL-6 and age when predicting somatic complaints, *b* = 0.01, *SE* = 0.01, *p* = 0.10. Specifically, IL-6 was associated with more somatic complaints among older participants (>66 years), *b* = 0.46, *SE* = .13, *p* < 0.001, and average aged participants (41–66 years), *b* = 0.32, *SE* = 0.10, *p* = 0.001, but not younger participants (<41 years), *b* = 0.18, *SE* = 0.13, *p* = 0.16. Using the Johnson-Neyman technique, the association between IL-6 and somatic complaints was significant and positive after about age 46. There was also a non-significant interaction between IL-6 and age when predicting interpersonal problems, *b* = −0.003, *SE* = 0.002, *p* = 0.06. Specifically, IL-6 was associated with more interpersonal problems among younger participants (<41 years), *b* = 0.11, *SE* = 0.04, *p* = 0.003, average aged participants (41–66 years), *b* = 0.06, *SE* = 0.03, *p* = 0.02, but not older participants (>66 years), *b* = 0.02, *SE* = 0.04, *p* = 0.59. Using the Johnson-Neyman technique, the association between IL-6 and greater interpersonal problems was significant and positive up to about age 56.

## Discussion

**Figure 3:**
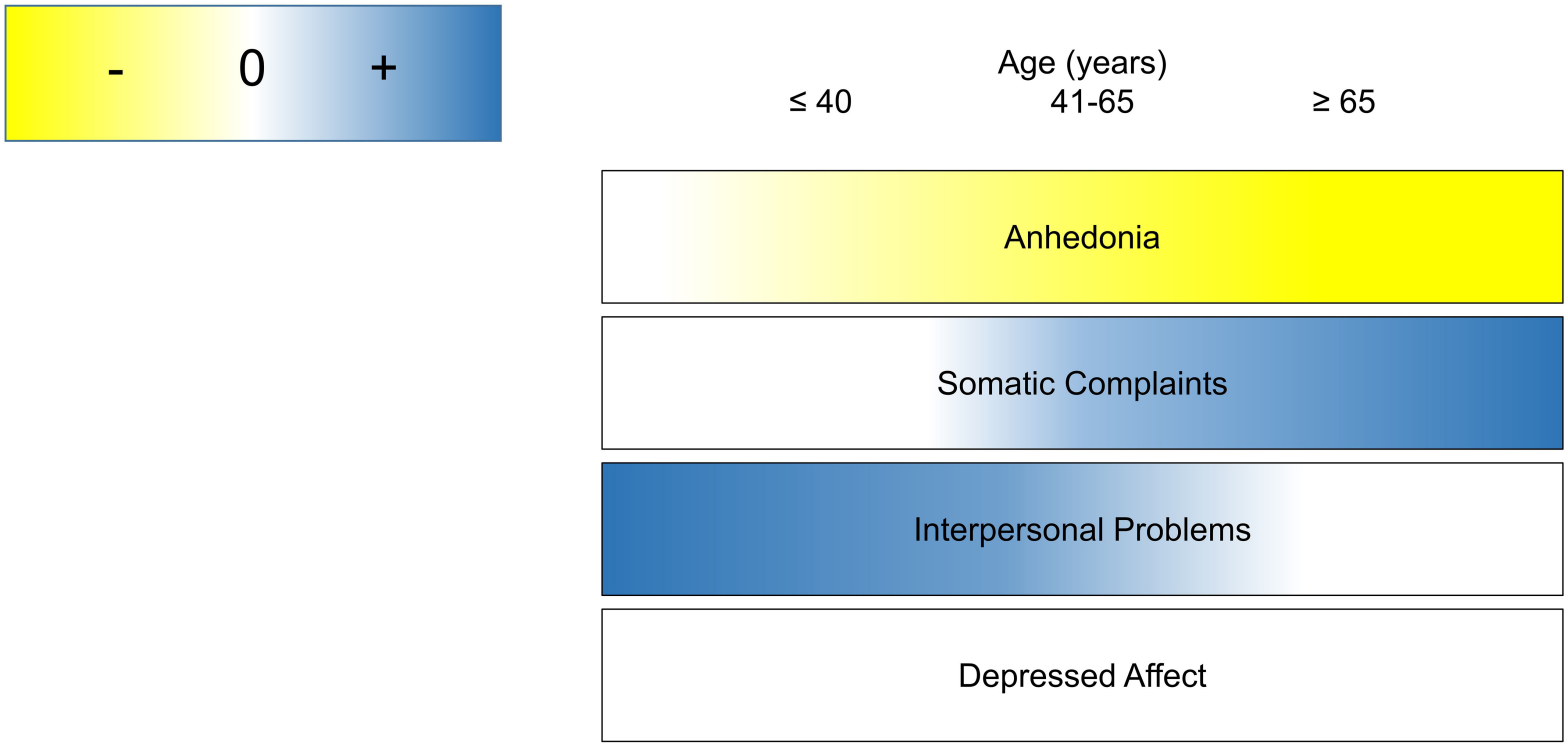
Conceptual summary of age as a context for the role of inflammation in depressive symptom domains.

The complex relationship between aging, inflammation and depressive symptoms may reflect a dynamic developmental process by which the brain becomes differentially susceptible to the behavioral effects of inflammation. In this large, community sample, the association between inflammation and depressive symptoms varied as a function of age and domain of impairment, particularly with respect to anhedonia and somatic complaints. Specifically, inflammation was associated with more reported somatic complaints and less anhedonia as age increased. We observed this association most robustly using CRP as an inflammatory biomarker and observed similar, but non-significant patterns using IL-6. A conceptual figure summarizing the observed associations between inflammation and each symptom domain as a function of age is shown in Figure 3. These findings have important implications for developmental immunology and neuroscience, as well as the conceptualization of somatic complaints among older adults.

More CRP was associated with less anhedonia in our sample, an effect which grew in magnitude with age. The present findings provide promising preliminary evidence that aging is an important context to consider in the role of inflammation in anhedonia, the neural mechanisms of which warrant further investigation. Indeed, in a study conducted with another subset of MIDUS participants, higher inflammation was associated with lower limbic reactivity to positive affective images and greater connectivity between the limbic system and prefrontal cortex (77). Inflammation affects reward learning, sensitivity, and motivation through frontostriatal circuit function in many ways, including interfering with dopamine synthesis (78). Additionally, the brain undergoes many changes across the lifespan, including decreases in white matter, gray matter, the volume and structural integrity of the striatum, a critical reward related region, as well as a reduction in dopaminergic receptors (56, 79–81), and declines in the frontostriatal circuit (82, 83). Inflammation may have a disproportionate impact on reward processing depending on the structural and functional integrity of reward circuits in the brain. In the current study, the negative association between CRP and anhedonia was somewhat unexpected given previous studies showing that inflammation can attenuate positive affect and psychological processes associated with reward (42–44, 84, 85). Yet, mild immune activation with acute laboratory stress or a vaccine can increase reward learning and motivation in the short-term (86, 87). The complexities of reward processes, which are comprised of learning, motivation, and sensitivity, must also be considered when thinking about how reward neurocircuitry relates to self-reported symptoms of anhedonia. The anhedonia subscale on the CES-D corresponds to subjective positive appraisals of one's life, happiness, and worth, which may more accurately reflect higher order psychological states than transient positive emotions measured in many studies (88). It is also possible that our negative association between inflammation and anhedonia was influenced by the very low incidence of anhedonia in our sample, and thus this finding should be interpreted with caution until it can be replicated within a clinical sample.

There was a stronger association between inflammation, as measured by CRP, and somatic complaints with increasing age. Of note, we observed this sensitivity consistently among individuals over the age of 51 for CRP, with a similar but non-significant inflection point using IL-6. Importantly, this interaction was significant after accounting for the unexpected, inverse association between age and somatic complaints. Somatic complaints on the CES-D include an increase in irritability, decrease in appetite, trouble with concentration, fatigue, sleep difficulty, and lack of motivation. Normative changes in structure within the aging brain may explain an increasing sensitivity of aging individuals to inflammation in relation to somatic complaints. This may also involve dopaminergic processes because inflammation interferes with the synthesis, transmission, and re-uptake of dopamine (89, 90), which has profound implications for many psychological phenomena, including somatic complaints such as fatigue. Indeed, the most consistent causal evidence linking inflammation to depressive endophenotypes exists for somatic complaints such as fatigue, sleep disturbance, and subjective cognitive difficulties (22). Studies examining correlates between inflammation and the structure and function of neural circuits implicated in different endophenotypes of depression could further elucidate how the context of aging affects the relationship between inflammation and depressive symptom domains.

Finally, it is important to note that we did not observe a link between inflammation and depressed affect in our sample, which is consistent with prior literature (42, 77, 91, 92) but often neglected in research on the role of inflammation in depressive symptoms. To our knowledge, the only studies which have found a significant link between inflammation and depressed affect have involved presenting the immune system with a short-term, strong inflammatory challenge which produces brief, high levels of inflammation in some individuals (22, 27, 30). Along these lines, it is also important to note that each of our models only accounted for between 3 and 5% of variance in symptoms. This is consistent with previous studies examining the association between inflammation and depressive symptoms (17, 93, 94). Depressive symptoms are largely heritable; with estimates ranging between 23 and 71% (95–98). Further, these models were based on observations within a community sample, not a clinical sample. These factors may have limited the between-subject variability to account for in our data. Whether inflammation accounts for more variance in symptom domains within clinical samples or after heritability is covaried out remains important to determine. Even so, characterizing the role of modifiable factors, such as inflammation, in health outcomes such as depression is critical to intervention development and healthcare practices.

### Future Directions

Normative changes in the structure and function of the human brain with increasing age may mitigate or exacerbate the effect of inflammation on some affective and behavioral outcomes. In addition to the increasingly negative association between inflammation and anhedonia, we observed a non-significant trend that the link between inflammation and interpersonal problems weakened among older individuals. These findings may indicate a reduced susceptibility to some of the detrimental effects of systemic inflammation with age. Normative age-related changes in the function and structure of the brain are complemented by the many psychological and social resources that continue to amass throughout the lifespan which may further buffer older individuals against the deleterious influence of inflammation on emotional well-being. For example, older adults experience less limbic activation when presented with negative stimuli and recall positive memories more easily than negative memories (99, 100).

One strength of this study was its use of a large, epidemiological sample with participants from two nations (USA and Japan). Given the diversity of the combined samples, these observations may generalize across a wide range of individuals and societies which will be exciting to explore. However, the methodological differences in recruitment and data collection between the studies (MIDJA, MIDUS2, MIDUS-R) impeded our ability to account for the role of racial or ethnic background above and beyond the study from which the data was drawn. For example, it is possible that the role of aging in the association between inflammation and depressive symptom domains may differ between Asian and Caucasian individuals, yet any variable indicating race or ethnicity would be entirely confounded by the study. While the CES-D has been validated as a measure of depression and depressive symptoms among Japanese adults (72), there is evidence that the factor structure of depressive symptoms using this measure differs from that observed in the U.S. such that the interpersonal problems subscale does not warrant its own symptom domain (73). It is also plausible and likely that the context of aging in immune to brain communications has different implications for our Japanese (MIDJA) and U.S. samples (MIDUS2 and MIDUS-R). Cross-cultural comparisons between aging Japanese persons and aging Americans have shown that both of these groups report similar levels of increased well-being with age (101). It is also noteworthy that our study found a non-significant trend between IL-6 and interpersonal problems in our combined sample, even though IL-6 was lower in the MIDJA sample compared to the MIDUS sample overall (102). To our knowledge, no studies have examined neurodevelopmental or aging-related inflammatory differences between American and Japanese samples. These studies would be a valuable follow-up to the present findings.

### Limitations

These results should be considered in the context of the study's limitations. First and foremost, these data are cross-sectional and cannot be used to infer causality. Our model focuses on the influence of inflammation on depressive symptoms, although the relationship between inflammation and depression can be characterized as bidirectional (93, 94, 103, 104). Longitudinal studies can provide relevant information to inform future interventions. Second, it is important to note that our outcome measure was depressive symptoms rather than depressive episodes examined within a community sample where the prevalence of major depression was likely low. Yet, functional impairment increases linearly with depressive symptoms, even at subthreshold levels and particularly in the elderly, underscoring the clinical utility of understanding the pathogenesis of depressive symptoms regardless of diagnostic status (49, 50, 105, 106). Nevertheless, replication within clinical or treatment-seeking samples is needed, particularly in a sample with elevated somatic complaints. With respect to measurement of different domains of depressive symptoms, each domain differed in its potential sensitivity. Specifically, the interpersonal problems subscale of the CES-D is only comprised of 2 items, which may have limited our ability to detect associations with this construct.

Further, inflammation is a dynamic and multifaceted biological process that can be measured at the intracellular, cellular, molecular, and neural levels. In the present study, we have focused only on two common circulating markers of inflammation, which yielded similar patterns. However, there were only reliable associations with CRP not IL-6. One possible explanation for this observation in our data was that IL-6 appeared to increase with age while CRP did not (see Table 1), leaving more potential variance for CRP to explain in our depressive symptom domains. The more robust association between CRP, relative to IL-6, and depressive symptoms is consistent with observations in longitudinal studies of depression (19), though the broader literature shows a consistent association between IL-6 and depression as well (12). This particular distinction between CRP and IL-6 may or may not be meaningful. CRP and IL-6 are related inflammatory markers, as CRP is stimulated by IL-6. Clinically speaking, CRP is already used in other fields of medicine which increases its potential for adoption in psychiatry, and it is already being used to inform depression treatment [c.f. (107)]. Examination of whether age moderates the association between other inflammatory markers and depressive symptoms is needed. This will be particularly important in future work that takes the source of inflammation into consideration. In the present study, we were somewhat agnostic to the cause of inflammation (e.g., stress, injury, illness), though many inflammatory proteins have specific signaling functions that most certainly vary in their influence on the central nervous system. Finally, the moderating role of age in the link between inflammation and different depressive symptom domains may indicate that age is a proxy for several developmental processes. Here, we focused on the combined, non-specific biological and psychosocial developmental processes that occur with chronological age, but these phenomena may not unfold along the same time course and may be interdependent.

### Conclusions

Inflammation is a well-established causal mechanism in the development of many depressive symptoms (8, 13, 22). Yet, little attention has been given to the stability of this phenomenon across the human lifespan. Given our vast and growing knowledge of how the human brain changes across the lifespan, there are likely to be periods of greater susceptibility and periods of relative protection from these putative effects. These preliminary observations suggest that the association between inflammation and somatic complaints is stronger, while the association between inflammation and anhedonia is weaker, among older adults. If corroborated with experimental evidence, these findings may have important implications for the conceptualization of somatic complaints among older adults within healthcare settings. Indeed, individuals above the age of 65 are disproportionately diagnosed with high rates of inflammatory physical disorders and low rates of depression (54). At the same time, older adults are also more likely to die by suicide than younger adults [see (51) for a special issue on this topic; (52, 53)]. It is possible that treatment-seeking older adults with somatic complaints could benefit from some of the well-established treatments for depression. These patients may be exhibiting a differential presentation of depressive symptoms than their younger counterparts. Indeed, there is evidence that the same pharmacological treatments most effective for treating depressive symptoms also provide relief for somatic symptoms experienced by older adults (108, 109). Our preliminary observations may inform important modifiable factors that can be used for novel pharmacological and behavioral intervention development for older adult populations.

## Data Availability Statement

Publicly available datasets were analyzed in this study. This data can be found here: https://doi.org/10.3886/ICPSR29282.v9; https://doi.org/10.3886/ICPSR36901.v6; https://doi.org/10.3886/ICPSR34969.v4. For more information, you can visit the MIDUS Study website: http://www.midus.wisc.edu/.

## Ethics Statement

The studies involving human participants were reviewed and approved by the University of California Los Angeles IRB, University of Wisconsin IRB, Georgetown University IRB, and the University of Tokyo IRB. The participants provided their written informed consent to participate in this study.

## Author Contributions

KS and KK contributed conception and design of the secondary data analysis. M-LT retrieved and organized the database. KK conducted the data analyses and interpretation of the data. All authors contributed to the writing and revision of the manuscript.

## Conflict of Interest

The authors declare that the research was conducted in the absence of any commercial or financial relationships that could be construed as a potential conflict of interest.
